# Passive Screening for Depressive Symptoms Using Daily Wrist Actigraphy and Deep Learning: Model Development and Validation Study

**DOI:** 10.2196/91479

**Published:** 2026-07-15

**Authors:** Doljinsuren Enkhbayar, Somin Oh, Jinhee Lee, Min-Hyuk Kim, Erdenebayar Urtnasan, Jaehong Key

**Affiliations:** 1 Department of Biomedical Engineering, Mirae campus, Yonsei University, 1, Yeonsedae-gil, Heungeop-myeon, Wonju-si, Gangwan-do, 26493, Republic of Korea, 82 33 760 5270, 82 33 760 2919; 2 Department of Psychiatry, Wonju College of Medicine, Yonsei University Mirae Campus, Wonju, Gangwon-do, Republic of Korea; 3 Division of AI Semiconductor, Yonsei University Mirae Campus, Wonju, Gangwon-do, Republic of Korea

**Keywords:** actigraphy, wearable devices, depressive symptoms, depression screening, artificial intelligence, deep learning, passive monitoring

## Abstract

**Background:**

Depressive symptoms are common yet often underrecognized in routine care, underscoring the need for scalable screening approaches beyond episodic self-report assessments. Wearable actigraphy can passively and continuously capture daily activity and 24-hour rest–activity rhythms associated with depressive symptom burden. However, the performance of artificial intelligence (AI) models that leverage actigraphy data for depressive symptom screening remains insufficiently established.

**Objective:**

This study aimed to develop and evaluate AI-based models for passive screening of depressive symptoms from daily wrist actigraphy data.

**Methods:**

We analyzed actigraphy recordings from 1160 Hispanic/Latino adults in the Hispanic Community Health Study/Study of Latinos who completed the 10-item Center for Epidemiologic Studies Depression scale (CESD-10), a self-reported depressive symptom screening scale. Multichannel actigraphy data, including activity counts, light exposure, and wake status, were used as inputs to 5 deep learning architectures to classify CESD-10–defined depressive symptom groups, comparing mild and higher symptoms with the normal group.

**Results:**

Actigraphy-derived behavioral markers differed across depressive symptom groups, showing lower daytime activity and altered circadian rest–activity organization with increasing symptom burden. Among the 5 deep learning architectures evaluated, the long short-term memory model consistently demonstrated the strongest overall discrimination. In held-out testing, the long short-term memory model achieved a macro-averaged area under the receiver operating characteristic curve of 0.80, with the strongest discrimination observed for the higher depressive symptom group (area under the receiver operating characteristic curve 0.889). These findings indicate improved model discrimination with increasing symptom severity, although false-positive rates remained notable across both classification tasks.

**Conclusions:**

Our study suggests that actigraphy-derived data can support AI-based classification of depressive symptoms. An actigraphy-based AI model may serve as a scalable, passive, and noninvasive complementary signal to aid early screening alongside traditional depressive symptom assessments before clinical diagnosis.

## Introduction

Depression is a highly prevalent and debilitating psychiatric condition globally, with its burden increasing steadily in recent decades [1]. Despite its substantial impact on individuals and society, the mechanisms underlying depression remain complex and incompletely understood. Clinically, depression encompasses a broad spectrum of psychological, behavioral, and somatic symptoms, such as fatigue, sleep disturbances, persistent low mood, changes in appetite, anhedonia, and weight changes, impaired concentration, low self-esteem, and suicidal ideation [2,3]. These manifestations can markedly impair daily functioning, interpersonal relationships, and self-care [4].

Depression nevertheless remains underdiagnosed and undertreated in routine clinical practice, representing a critical gap in mental health care [2]. Many individuals are not identified during standard assessments, and screening is reported to occur in only 4.2% of adults without a prior diagnosis [5]. The nonspecific nature of depressive symptoms, which frequently overlap with somatic complaints such as fatigue or pain, can lead to misdiagnosis or attribution to other medical conditions [6,7]. Consequently, a substantial proportion of affected individuals remain untreated, increasing the likelihood of persistent symptoms and long-term functional impairment.

Given these challenges, there is increasing interest in objective behavioral markers that can complement traditional self-report tools [8]. Sedentary behavior, reduced physical activity, and sleep disruption have been consistently linked to depressive symptoms [9]. However, many studies rely primarily on self-reported measures, which are susceptible to subjectivity and limitations in memory accuracy. In contrast, wearable devices can passively and continuously monitor activity and sleep, providing an objective approach to capturing behavioral changes relevant to depressive symptoms [10].

Passive monitoring modalities differ in their technical characteristics, the type of behavioral information captured, and their degree of standardization. Consumer wearables, such as Fitbit devices [11] or the Apple Watch [12], rely on proprietary, closed-source algorithms that limit methodological transparency and reproducibility. Smartphones capture behavior indirectly through usage-based proxies, such as screen time, GPS mobility [13], and call logs [14], which are susceptible to device heterogeneity and user-specific confounds. Research-grade actigraphy, by contrast, uses validated, open-standard triaxial accelerometry with well-characterized signal processing pipelines, making it particularly suitable for controlled cohort studies and systematic architectural model comparison.

Actigraphy, based on wrist-worn triaxial accelerometers, provides an unobtrusive and continuous method for monitoring physical activity, rest-activity cycles, and light exposure over extended periods [15]. It can reliably estimate sleep-wake patterns and behavioral rhythms, enabling the characterization of reduced activity levels, increased sedentary time, and irregular sleep-wake schedules commonly observed among individuals with elevated depressive symptoms [16]. Evidence from meta-analyses indicates that depressive symptoms are associated with lower overall activity, poor sleep efficiency, longer sleep-onset latency, greater wake after sleep onset, and attenuated circadian amplitude compared with the normal group [17]. Collectively, these actigraphy-derived measures complement questionnaires and clinical interviews by providing a quantitative representation of circadian dysregulation and reduced activity. They may also support more effective screening of depressive symptoms than self-report alone [18].

Recent advances in artificial intelligence (AI) have expanded the utility of actigraphy for screening depressive symptoms [19]. Prior work has ranged from conventional machine learning models trained in manually engineered features, such as step-derived activity summaries and circadian indices, to end-to-end deep learning approaches [20]. While feature-based methods, including gradient-boosted ensembles, can yield moderate performance, they may be less effective at capturing time-resolved structure in actigraphy signals and can be susceptible to overfitting when many correlated predictors are included [21]. Accordingly, deep learning models have been applied directly to raw or minimally processed actigraphy sequences. Existing work has examined convolutional and recurrent architectures [22], including long short-term memory (LSTM) and gated recurrent unit (GRU) models, alongside transformers, Gramian angular field–based convolutional neural networks (CNNs), and mixed-input frameworks that incorporate sleep-stage features or questionnaire data, reporting area under the curve (AUC) values ranging approximately between 0.68 and 0.94 [20]. However, many studies have relied on small or selected samples, focused on extreme symptom groupings, or required additional clinical inputs, limiting generalizability and scalability [23]. Moreover, most studies evaluate a single architecture, which restricts comparative insight into sequence-modeling strategies [24]. Therefore, systematic comparisons of end-to-end deep learning models trained on multichannel actigraphy in large, ethnically diverse cohorts using validated self-report screening tools remain needed.

Despite these advances, actigraphy-based approaches for depressive symptoms have shown modest and variable performance, with reported accuracy and AUC values spanning a wide range across studies [25]. Much of the prior work has been developed using relatively small or demographically narrow samples, which increases the risk of overfitting and may limit the generalizability of findings to broader populations. Moreover, both machine learning and deep learning models can be difficult to interpret, particularly when complex architectures or extensive feature engineering are used, making it challenging to relate predictions to meaningful behavioral or physiological patterns [26]. Limited interpretability can hinder clinical uptake, as clinicians may be reluctant to act on algorithmic outputs without a clear connection to recognizable patterns in patients’ daily activities and sleep. Accordingly, there is a need for actigraphy-based screening models that achieve robust discrimination, with transparency and clinical interpretability identified as important directions for future development [27].

We developed and evaluated an AI-based screening approach that leverages wrist-worn actigraphy to identify depressive symptoms, using the 10-item Center for Epidemiologic Studies Depression scale (CESD-10) as the reference screening tool. We trained and compared 5 deep learning architectures on 7 days of multichannel actigraphy time series to classify CESD-10 depressive symptoms. We hypothesized that a higher symptom burden would be reflected in characteristic behavioral signatures, including reduced overall activity and disrupted rest-activity rhythms, which sequence models could learn from raw actigraphy patterns. This study evaluates whether daily actigraphy-based AI models can support objective and unobtrusive screening for depressive symptoms.

## Methods

### Study Design and Participants

**Figure 1.**
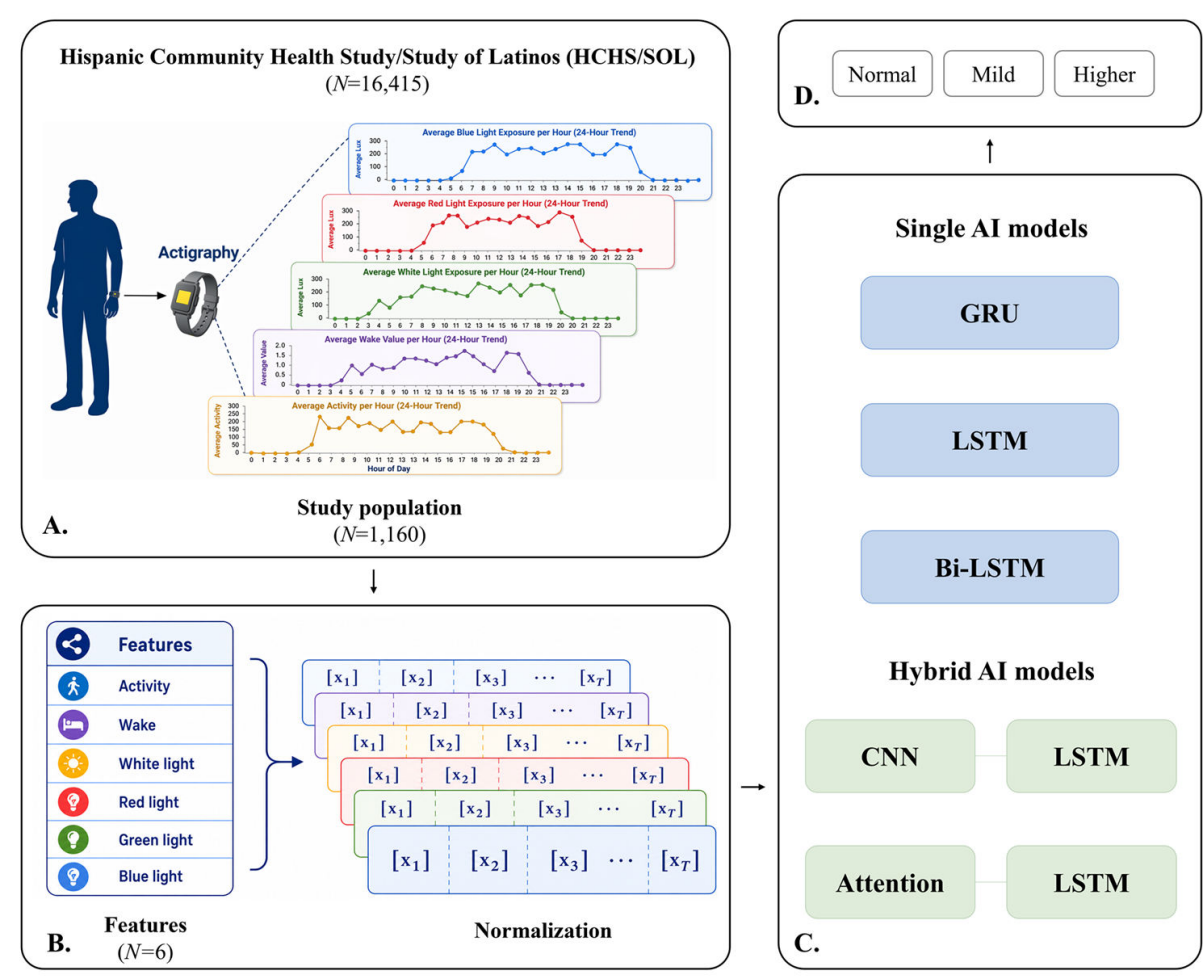
Overview of the methodological framework for screening depressive symptoms using actigraphy-derived data. (A) Study design and participants, (B) data processing and feature extraction, (C) artificial intelligence–based models, and (D) output. AI: artificial intelligence, Bi-LSTM: bidirectional long short-term memory, CNN: convolutional neural network, GRU: gated recurrent unit, LSTM: long short-term memory.

The Hispanic Community Health Study/Study of Latinos (HCHS/SOL) is a multicenter, population-based cohort study that enrolled 16,415 noninstitutionalized Hispanic/Latino adults aged 18 to 75 years across 4 US field centers in Miami, San Diego, the Bronx, and Chicago. Baseline examinations from 2008 to 2011 included standardized assessments of sociodemographic characteristics, lifestyle factors, medical comorbidities, and cardiometabolic biomarkers [28]. The Sueño ancillary study recruited 2087 adults aged 18‐65 years from the parent cohort for actigraphy-based assessment of sleep and circadian rhythms (Figure 1A).

In the parent HCHS/SOL Sueño study, exclusion criteria included pregnancy, cognitive dysfunction affecting reliable self-report or informed consent, and severe mobility limitations such as hemiparesis or quadriparesis. Actigraphy was collected over 7 days using an Actiwatch Spectrum device (Philips Respironics) worn on the nondominant wrist to continuously record activity counts, multiwavelength light exposure, and sleep-wake status. After standardized quality-control procedures that excluded recordings with prolonged off-wrist intervals or implausible signal patterns, valid actigraphy data were available for 1887 participants. We then excluded 217 participants with missing demographic or psychometric data, yielding 1670 participants eligible for propensity score matching. Using a 1:1 propensity score matching approach matched on age, sex, and BMI, we derived a final analytic cohort of 1160 participants (71.4% female, aged 18‐65 years, BMI 21.9-41.2 kg/m²), comprising 580 participants with normal depressive symptom scores and 580 with depressive symptoms. The depressive symptom group was further classified into mild and higher subgroups based on CESD-10 scores (Figure S1 in Multimedia Appendix 1).

All dataset partitioning and model evaluation were performed at the participant level. The matched cohort was then partitioned into training (70%, n=812), validation (10%, n=116), and test (20%, n=232) sets, with no participant appearing in more than one split. A fixed hold-out design was selected over cross-validation because the sequential, time-structured nature of actigraphy data makes random k-fold partitioning susceptible to temporal leakage across participants. The large number of epoch-level training samples (>17 million) per channel further mitigates overfitting risk despite the moderate participant count. Epoch-level counts for each channel are provided in Table 1 for descriptive purposes only; all model development and performance evaluations were conducted at the participant level.

Data access, documentation, and governance were provided through the National Sleep Research Resource (NSRR) under a controlled-access data use agreement. Institutional review board approvals were in place at all participating sites, and written informed consent was obtained from all participants.

**Table 1.** Total epoch counts for each actigraphy channel across the training, validation, and test splits.

Features	Training set	Validation set	Test set	Total
Activity	17,336,985	2,487,580	4,898,740	24,723,305
Wake	17,325,681	2,486,083	4,895,117	24,706,881
White light	17,335,426	2,487,011	4,898,349	24,720,786
RGB^a^ light	17,336,985	2,487,580	4,898,740	24,723,305
Total	69,335,077	9,948,254	19,590,946	98,874,277

a R: red, G: green, and B: blue light channels have an equal number of epochs.

### Depressive Symptoms Assessment

Depressive symptoms were measured using the CESD-10, a validated self-report screening tool widely used in epidemiologic and community-based studies [29]. Items are rated on a 4-point response scale from 0 to 3, yielding a total score from 0 to 30, with higher scores indicating greater depressive symptom burden over the past week [30]. Following prior HCHS/SOL analyses, participants with CESD-10 scores of 10 or higher were classified as having elevated depressive symptoms [31]. For the present analyses, CESD-10 scores were further categorized into three groups to capture graded symptom burden: 0 to 9 (normal), 10 to 14 (mild), and ≥15 (higher depressive symptoms). These categories were used to describe baseline characteristics and to evaluate trends in sociodemographic, behavioral, and clinical factors across increasing symptom severity. CESD-10 has demonstrated good psychometric properties across diverse populations, supporting its use as a community-based screening measure for depressive symptoms [32].

### Data Processing and Feature Extraction

**Figure 2.**
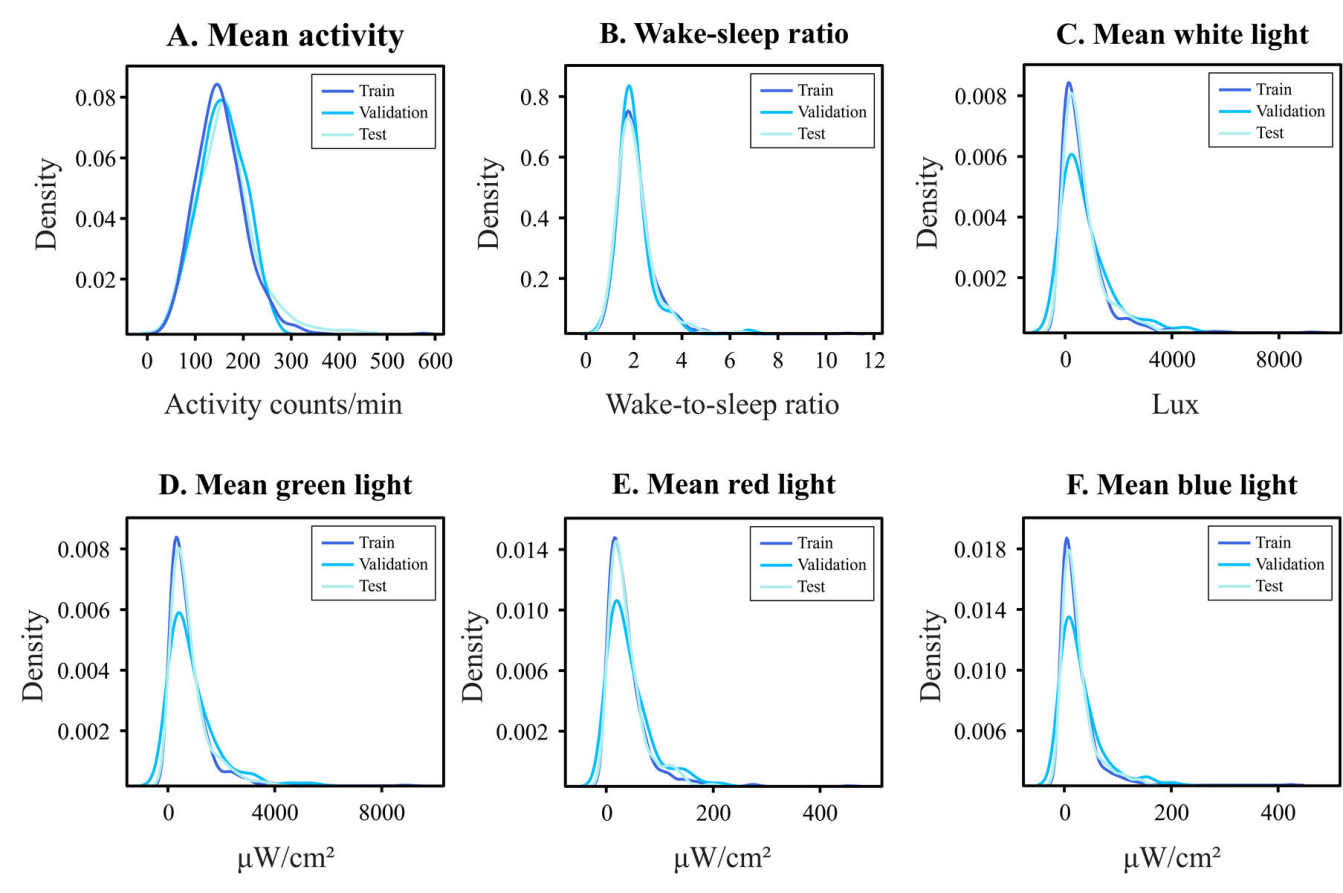
Density distributions of *z* score–normalized actigraphy features across dataset splits. Kernel density curves for the training, validation, and test sets show substantial overlap, indicating comparable feature distributions across splits and supporting consistent data representation during model development and evaluation. (A) Mean activity, (B) wake-to-sleep ratio, (C) mean white light, (D) mean green light, (E) mean red light, and (F) mean blue light.

Raw wrist actigraphy recordings were preprocessed to support downstream model development and evaluation. For each participant, signals were segmented into 30-second epochs containing activity counts, binary wake status, and light exposures in the white, red, green, and blue channels. Epochs flagged as invalid or off-wrist under the HCHS/SOL quality control criteria were removed before analysis. After data cleaning, the remaining time series were standardized using *z* score normalization. To prevent information leakage, the mean and SD were estimated using the training set only and applied unchanged to the validation and test sets for consistent scaling across splits (Figure 1B). The normalized sequences were segmented into nonoverlapping windows of 2000 consecutive epochs, corresponding to approximately 16.7 hours, providing fixed-length inputs for the deep learning architectures. The resulting inputs comprised the following six synchronized channels: activity (Figure 2A), wake status (Figure 2B), white light exposure (Figure 2C), green light exposure (Figure 2D), red light exposure (Figure 2E), and blue light exposure (Figure 2F). Across the training, validation, and test splits, this yielded approximately 24 to 25 million 30-second epochs per channel in total (Table 1). Minor differences in epoch counts across channels reflect channel-specific quality-control exclusions applied independently during preprocessing. Overall epoch proportions across splits remained consistent with the participant-level 70/10/20 allocation. This segmentation approach provided a large and representative set of training samples while maintaining consistent preprocessing across datasets and minimizing the risk of distributional shifts introduced by data handling.

To assess consistency across dataset splits, we examined the distributions of key actigraphy-derived features within the training, validation, and test sets (Figure 2). The *z* score–normalized distributions showed substantial overlap, indicating comparable feature profiles across splits and supporting the appropriateness of the partitioning. This consistency helps preserve statistical comparability between datasets and strengthens the reliability of model development and evaluation.

In addition to the raw actigraphy time series used for deep learning, we computed summary measures to describe 24-hour rest–activity rhythms for descriptive analyses and between-group comparisons. For each valid day, mean activity counts were calculated within eight 3-hour clock-time intervals spanning midnight to 2:59 AM through 9 PM to 11:59 PM and then averaged across monitoring days. Using the 30-second activity signal, we quantified circadian rhythm metrics, including intradaily variability, interdaily stability, and relative amplitude derived from the most active 10-hour window (M10) and the least active 5-hour window (L5), along with the onset times of M10 and L5. We also fit a 24-hour cosinor model to each participant’s activity profile to estimate the midline statistic of rhythm, log-transformed amplitude, and acrophase. For each metric, values were aggregated across valid days to obtain a participant-level estimate, which was compared across CESD-10 depressive symptom categories.

### AI-Based Models

We implemented 5 deep learning architectures to classify depressive symptoms using multichannel wrist actigraphy data, including a GRU [33], LSTM [34], bidirectional LSTM (Bi-LSTM) [35], 1-dimensional convolutional neural network–long short-term memory (CNN-LSTM) [36], and attention-LSTM [37] (Figure 1C). These architectures were selected to represent diverse sequential modeling strategies, ranging from conventional recurrent networks to bidirectional and hybrid designs, thereby enabling a systematic comparison of their ability to capture temporal dependencies in multichannel actigraphy data. Each model leveraged different modeling strengths, from local feature extraction and long-range temporal dependency learning to bidirectional sequence modeling, enabling identification of the most effective approach for classifying depressive symptom severity from wrist-worn sensor data.

All models received input sequences of 2000 time steps with 6 synchronized channels, including activity counts, wake status, and multiwavelength light exposure (white, red, green, and blue), and produced a 3-class probability distribution through a softmax activation function. A dropout rate of 0.3 was selected based on prior LSTM-based studies [34] and preliminary validation experiments comparing rates of 0.2, 0.3, and 0.5, with 0.3 yielding the best validation performance. The final class prediction was determined by selecting the class with the highest softmax probability (Table 2).

**Table 2.** Summary of deep learning model architecture and training configurations. All models were trained using the AdamW optimizer with a mini-batch size of 32, focal loss with class-specific weighting, and a Cosine Annealing Warm Restarts learning rate scheduler.

Model	Key components	Units or filters	Learning rate	Max epochs (early stop)	Total parameters
Single AI^b^ models					
GRU^f^	4 GRU layers + BN^a^ + Dense	60‐120 units	3×10⁻⁴	150 (patience=30)	170,345
LSTM^c^	4 LSTM layers + BN + Dense	60‐120 units	3×10⁻⁴	150 (patience=30)	258,585
Bi-LSTM^d^	4 Bi-LSTM layers + BN + Dense	60‐120 units (bidirectional)	3×10⁻⁴	150 (patience=30)	518,065
Hybrid AI models					
CNN-LSTM^e^	3 Conv1D + MaxPooling + 2 LSTM + Dense	32‐128 filters, 64 LSTM units	3×10⁻⁴	150 (patience=30)	86,817
Attention LSTM	3 Conv1D + MaxPooling + Attention + LSTM + Dense	32‐128 filters, 64 LSTM units, 128d attention	3×10⁻⁴	150 (patience=30)	136,097

a BN: batch normalization.

b AI: artificial intelligence.

c LSTM: long short-term memory.

d Bi-LSTM: bidirectional long short-term memory.

e CNN-LSTM: convolutional neural network–long short-term memory.

f GRU: gated recurrent unit.

### Single AI Models

The GRU, LSTM, and Bi-LSTM models were implemented using 4 stacked recurrent layers with 60‐120 hidden units, followed by batch normalization and a fully connected output layer (Table S2 and Figures S2-S3 in Multimedia Appendix 1). GRU and LSTM models use different gating formulations but are both widely applied to sequential physiological signals. In contrast, the Bi-LSTM processes each sequence in both forward and backward directions, allowing the model to integrate information from both earlier and later time points within the same window (Table S3 and Figure S6 in Multimedia Appendix 1).

### Hybrid AI Models

The CNN-LSTM model consisted of three 1D convolutional blocks with filter sizes of 32‐128, each followed by rectified linear unit activation and max-pooling, and 2 LSTM layers with 64 hidden units (Table S4 and Figure S7 in Multimedia Appendix 1). The attention-LSTM extended this architecture with a single-head attention module before the LSTM layer (Table S5 and Figure S8 in Multimedia Appendix 1).

### Model Training

All models were trained for up to 150 epochs using a mini-batch size of 32, with early stopping applied for 30 consecutive epochs. A Cosine Annealing Warm Restarts learning rate scheduler was applied to facilitate convergence. Hyperparameters, including learning rate (3×10⁻⁴), hidden units, and dropout rate, were selected through a structured manual search over predefined ranges, with final configurations chosen to maximize validation AUC. To further optimize classification performance, threshold calibration was performed on the validation set using the Nelder-Mead optimization method to maximize macro *F*_1_, with calibrated thresholds applied during test set evaluation.

### Class Imbalance

The training cohort reflected moderate class imbalance, with 580 participants classified as normal, 318 as mild, and 262 as higher depressive symptoms (Figure 1D). This was addressed using a combination of 3 strategies. First, sequence-level oversampling was applied to the training set, augmenting minority class samples with Gaussian jitter and random scaling until each class reached 65% of the majority class size (target ratio=0.65). Second, weighted random sampling was applied during mini-batch construction to further balance class representation. Third, focal loss with class-specific weights (mild: 1.8×, higher: 2.0× relative to normal) was used to penalize misclassification of minority classes during training.

### Experiments and Evaluations

All experiments were implemented in Python (version 3.11.4; Python Software Foundation), with model development and training carried out using the Keras API within TensorFlow 2.x [38]. Statistical analyses were carried out using SciPy (version 1.8.1; SciPy developers) [39], and machine learning metrics were computed using scikit-learn (version 1.1.2; scikit-learn developers) [40].

Training and evaluation were performed on a Windows 11 workstation equipped with an NVIDIA GTX 1080 Ti GPU. For efficient computation, the dataset was divided into minibatches of 256 samples. Model parameters were updated using gradient accumulation after each minibatch.

Model performance on the test set was summarized using standard classification metrics, including precision, specificity, sensitivity, accuracy, *F*_1_-score, and the area under the receiver operating characteristic curve (AUROC). To assess the robustness of model performance, 95% CIs for AUROC were estimated using bootstrap resampling (1000 iterations) on the test set. All metrics were computed from the test-set confusion matrix. Metric definitions are provided below [41]:

Precision = TP / (TP + FP)

Specificity = TN / (TN + FP)

Sensitivity (Recall) = TP / (TP + FN)

Accuracy = (TP + TN) / (TP + TN + FP + FN)

*F*_1_-score = 2 × (Precision × Recall) / (Precision + Recall)

For multi-class classification, accuracy was computed within a one-versus-rest framework, in which each depressive symptom group was designated as the positive class, and the remaining two groups were combined as the negative class. Per-class accuracy thus reflects the proportion of correctly classified instances, both true positives and true negatives, within each binary classification setting.

Here, TP represents depressed participants correctly classified as depressed, and TN represents nondepressed participants correctly classified as nondepressed. FP refers to nondepressed participants misclassified as depressed, whereas FN indicates depressed participants misclassified as nondepressed.

### Ethical Considerations

This study was a secondary analysis of deidentified wrist-worn actigraphy and associated covariate data from the HCHS/SOL, obtained through the NSRR. The original HCHS/SOL study received institutional review board approvals at all participating field centers (University of North Carolina at Chapel Hill, Albert Einstein College of Medicine, University of Illinois at Chicago, University of Miami, and San Diego State University), and written informed consent was obtained from all participants. The dataset analyzed in this study was deidentified before release in accordance with the Health Insurance Portability and Accountability Act and contained no direct or indirect personal identifiers. This study involved secondary analysis of existing deidentified data and did not involve recruitment of participants, interaction with participants, intervention, or access to identifiable private information. Because this study used only deidentified secondary data and did not involve human participants as defined under applicable institutional and national research ethics regulations, additional institutional review board approval and informed consent were not required for this secondary analysis. Data were accessed under an approved Data Access and Use Agreement with Brigham and Women's Hospital through the NSRR (NHLBI R24 HL114473). No attempt was made to identify or reidentify any participant. All data were fully deidentified; no identifiable participant information was accessed, and no identifying details appear in this study.

## Results

### Demographic Characteristics

A total of 1160 participants with valid baseline actigraphy and CESD-10 data were included. Based on CESD-10 scores, 580 (49.1%) participants were classified into the normal group (CESD-10 <10), 318 (27.9%) into the mild symptom group (CESD-10 =10‐14), and 262 (23%) into the higher symptom group (CESD-10 ≥15). Higher depressive symptom burden was associated with a greater prevalence of being unmarried, currently unemployed, and current smoking (all *P*<.001; Table 3).

**Table 3.** Baseline demographic and clinical characteristics stratified by depressive symptom group. Group differences were assessed using chi-square tests for categorical variables and one-way ANOVA for continuous variables, as appropriate.

Measure	Normal (n=580)	Mild (n=318)	Higher (n=262)	*P* value^a^
Demographic				
Sex, n (%)				.28
Female	405 (69.83)	226 (71.07)	197 (75.19)	
Male	175 (30.17)	92 (28.93)	65 (24.80)	
Age (years), n (%)				.39
18‐29	54 (9.31)	26 (8.18)	28 (10.69)	
30‐39	85 (14.66)	52 (16.35)	31 (11.83)	
40‐49	208 (35.86)	122 (38.36)	86 (32.82)	
50‐64	233 (40.17)	118 (37.11)	117 (44.66)	
BMI (kg/m²), mean (SD)	30.53 (6.20)	30.65 (6.63)	30.48 (6.20)	.96
Married, n (%)	138 (23.79)	109 (34.28)	93 (35.50)	<.001
Education, n (%)				.08
No HS^b^ or GED^c^	127 (39.94)	47 (30.72)	85 (32.44)	
≤HS or GED	106 (33.33)	61 (39.87)	103 (39.31)	
>HS or GED	85 (26.73)	45 (29.41)	74 (28.24)	
Unemployed	263 (45.34)	173 (54.40)	181 (69.08)	<.001
Lifestyle and behavioral, n (%)				
Smoker	97 (16.72)	83 (26.10)	91 (35.94)	<.001
Alcohol use	253 (43.62)	147 (46.23)	108 (40.44)	.48
Comorbidities, n (%)				
Hypertension	176 (30.34)	102 (32.08)	93 (35.27)	.33
Diabetes mellitus	106 (18.28)	71 (22.33)	54 (20.68)	.33
Cerebrovascular disease	12 (2.07)	11 (3.46)	6 (2.22)	.43
Coronary heart disease	37 (6.38)	18 (5.66)	19 (7.13)	.74
Chronic kidney disease	267 (46.11)	138 (43.81)	135 (52.03)	.14
Dyslipidemia	220 (38.00)	104 (33.02)	99 (33.44)	.24

a *P* values reflect between-group comparisons based on the tests described above.

b HS: high school.

c GED: general educational development.

In contrast, demographic characteristics and cardiometabolic comorbidities were comparable across depressive symptom groups, with no statistically significant differences observed in age, sex, education, BMI, alcohol use, diabetes, hypertension, cerebrovascular disease, chronic kidney disease, coronary heart disease, or dyslipidemia (all *P*>.05). In this community-based cohort, higher depressive symptom burden was more strongly associated with sociodemographic and behavioral factors, particularly marital status, unemployment, and smoking, than with cardiometabolic comorbidity. Specifically, unemployment exceeded two-thirds in the higher symptom group, and current smoking increased across depressive categories (Table 3).

### Daily Light Exposure and Rest Activity

Actigraphy-derived light exposure and rest-activity characteristics by depressive symptom groups are provided in Table 4. Time spent above 500 and 1000 lux did not differ significantly across groups, although values were numerically lower among participants with depressive symptoms. In contrast, mean daytime white-light exposure was reduced in the higher symptom group compared with the normal group.

**Table 4.** Wrist actigraphy–based light exposure, 24-h rest-activity, and clock-time movement indices across 10-item Center for Epidemiologic Studies Depression scale depressive symptom groups.

Feature	Normal (n=580)	Mild (n=318)	Higher (n=262)	*P* value
Daily light exposure, mean (SD)				
Avg bright ≥500 lux (min/day)	220.94 (176.09)	201.47 (162.08)	200.98 (164.43)	.05^a^
Avg bright ≥1000 lux (min/day)	163.90 (147.78)	146.44 (135.65)	145.60 (136.20)	.05
Mean daytime white light (lux)	1464.38 (1557.69)	1399.92 (1534.62)	1124.74 (1279.01)	.01
Nonparametric 24 h rest-activity, mean (SD)				
Intradaily variability	0.97 (0.1)	0.83 (0.2)	0.91 (0.3)	<.001
Interdaily stability	0.25 (0.14)	0.14 (0.15)	0.10 (0.14)	.05
M10^b^ activity	168.57 (51.71)	164.56 (63.10)	156.69 (52.79)	<.001
M10 onset	547.47 (122.05)	579.67 (127.29)	590.60 (126.52)	<.001
L5^c^ activity	12.62 (12.41)	14.78 (18.75)	15.92 (20.83)	<.001
L5 onset	341.43 (113.39)	309.39 (98.90)	289.69 (115.26)	<.001
Cosinor-based circadian rhythm, mean (SD)				
MESOR^d^	583.84 (195.76)	400.56 (186.32)	304.50 (172.49)	<.001
Log amplitude	4.84 (1.53)	4.76 (1.47)	4.56 (1.31)	.01
Acrophase	9.78 (3.73)	9.68 (4.17)	9.40 (3.20)	.01
Clock-time activity, mean (SD)				
Midnight-2:59 AM (midnight)	29.63 (34.60)	37.30 (41.85)	37.65 (37.70)	.01
3 AM-5:59 AM (predawn)	20.04 (26.53)	20.34 (26.97)	20.82 (28.04)	.05
6 AM-8:59 AM (early morning)	98.19 (66.03)	84.90 (57.21)	73.74 (53.92)	<.001
9 AM-11:59 AM (morning)	157.35 (68.08)	144.57 (70.02)	140.09 (60.05)	.01
Noon-2:59 PM (early afternoon)	170.09 (58.00)	170.68 (72.76)	156.42 (61.30)	.01
3 PM-5:59 PM (mid-afternoon)	161.72 (55.01)	162.35 (70.33)	152.04 (58.18)	.05
6 PM-8:59 PM (evening)	147.88 (51.90)	144.45 (58.38)	134.61 (58.43)	<.001
9 PM-11:59 PM (late-evening)	96.46 (47.25)	100.77 (54.39)	93.16 (56.49)	.05

a *P=*.05: statistically significant.

b M10: average activity within the most active 10-h period of the day.

c Average activity within the least active 5-h period of the day.

d MESOR: midline estimating statistics of rhythm.

Across nonparametric rest-activity measures, higher depressive symptom burden was associated with lower M10 activity and higher L5 activity, consistent with attenuation of the 24-hour rest-activity amplitude. M10 and L5 onset times were also shifted in symptomatic groups, indicating a modest change in the timing of the most and least active periods. Cosinor-derived metrics showed concordant patterns, with midline estimating statistics of rhythm and log-transformed amplitude decreasing from the normal to the higher depressive group, while acrophase occurred slightly earlier in participants with greater symptom burden. In contrast, interdaily stability and intradaily variability differed minimally between groups, suggesting that day-to-day regularity and fragmentation were largely preserved despite lower overall activity and reduced rhythmic amplitude.

### Time-of-Day Activity

**Figure 3.**
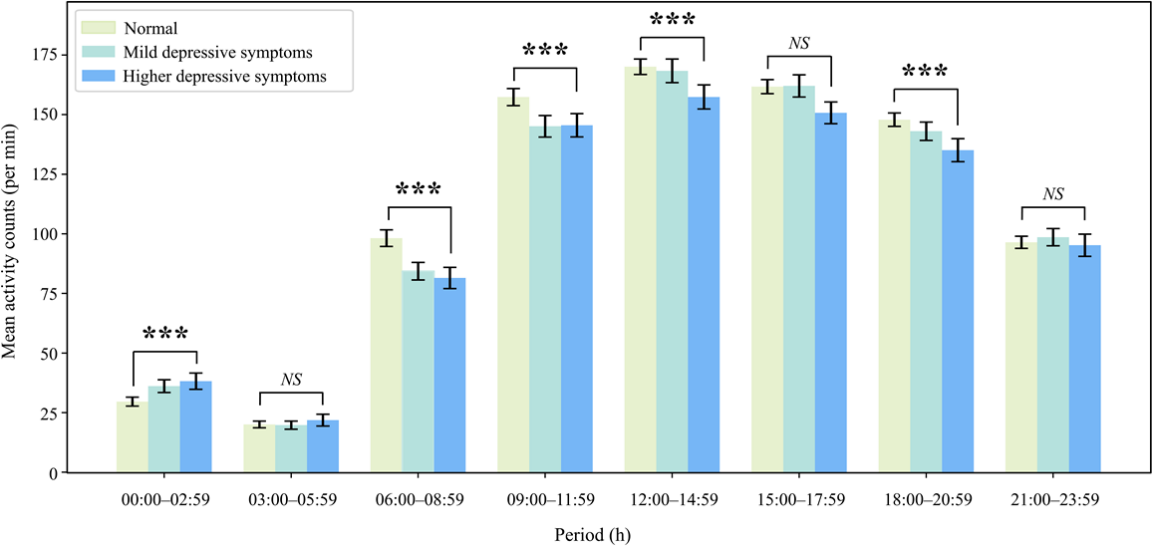
Mean activity counts across 3-h intervals by depressive symptom group. *P* values were calculated using unpaired 2-tailed student *t* tests (NS: not significant; ***P<.001).

Across the 24-hour day, mean activity declined as depressive symptom burden increased, with the largest separations observed during daytime periods (Figure 3). The higher-symptom group showed lower activity in the morning (6 AM-8:59 AM and 9 AM-11:59 AM), midday (noon to 2:59 PM), and early evening (6 PM-8:59 PM) relative to the normal group. In contrast, late-evening activity (9 PM-11:59 PM) and several late-night intervals differed only modestly between groups. Notably, activity between midnight and 2:59 AM was slightly higher in the higher-symptom group, suggesting a small redistribution of activity toward the nighttime period.

### AI Model Performance for Depressive Symptom Screening

Across the 5 deep learning architectures, we evaluated classification performance on the test set, with 95% CIs (Table 5). Among all models, discrimination was consistently higher for the higher depressive symptom group than for the mild group, suggesting that actigraphy-derived behavioral patterns may better differentiate higher symptom burden from normal. LSTM demonstrated the strongest overall performance, achieving the highest AUROC of 0.640 (95% CI 0.526‐0.736) for the mild depressive symptom group and 0.889 (95% CI 0.829‐0.942) for the higher depressive symptom group, followed by Bi-LSTM with a comparable AUROC of 0.881 (95% CI 0.817‐0.939). Model performance across the training, validation, and test sets is provided in Table S1 in Multimedia Appendix 1.

**Table 5.** Multiclass artificial intelligence model performance for depressive symptom screening.

Model	Normal (CESD^a^ <10)		Mild (CESD 10‐14)		Higher (CESD ≥15)	
	Accuracy^h^ (95% CI)	AUC^b^ (95% CI)	Accuracy^h^ (95% CI)	AUC^b^ (95% CI)	Accuracy^h^ (95% CI)	AUC^b^ (95% CI)
GRU^c^	0.634 (0.573-0.698)	0.811 (0.743-0.871)	0.639 (0.577-0.698)	0.557 (0.450-0.660)	0.786 (0.730-0.835)	0.777 (0.690-0.867)
LSTM^d^	0.726 (0.669‐0.778)	0.857 (0.798‐0.907)	0.682 (0.625‐0.738)	0.640 (0.526‐0.736)	0.850 (0.802‐0.895)	0.889 (0.829‐0.942)
Bi-LSTM^e^	0.625 (0.560‐0.681)	0.855 (0.791‐0.905)	0.577 (0.516‐0.637)	0.551 (0.443‐0.651)	0.854 (0.806‐0.899)	0.881 (0.817‐0.939)
CNN-LSTM^f^	0.597 (0.540‐0.661)	0.832 (0.772‐0.887)	0.630 (0.573‐0.690)	0.579 (0.460‐0.684)	0.758 (0.702‐0.810)	0.807 (0.731‐0.878)
Attention-LSTM^g^	0.706 (0.645‐0.758)	0.845 (0.785‐0.896)	0.646 (0.589‐0.702)	0.536 (0.432‐0.634)	0.859 (0.810‐0.903)	0.861 (0.785‐0.930)

a CESD: Center for Epidemiologic Studies Depression scale.

b AUC: area under the curve.

c GRU: gated recurrent unit.

d LSTM: long short-term memory.

e Bi-LSTM: bidirectional long short-term memory.

f CNN-LSTM: convolutional neural network–long short-term memory.

g Attention-LSTM: attention-based long short-term memory.

h Values are presented as point estimates with 95% confidence intervals (CI) in parentheses.

**Figure 4.**
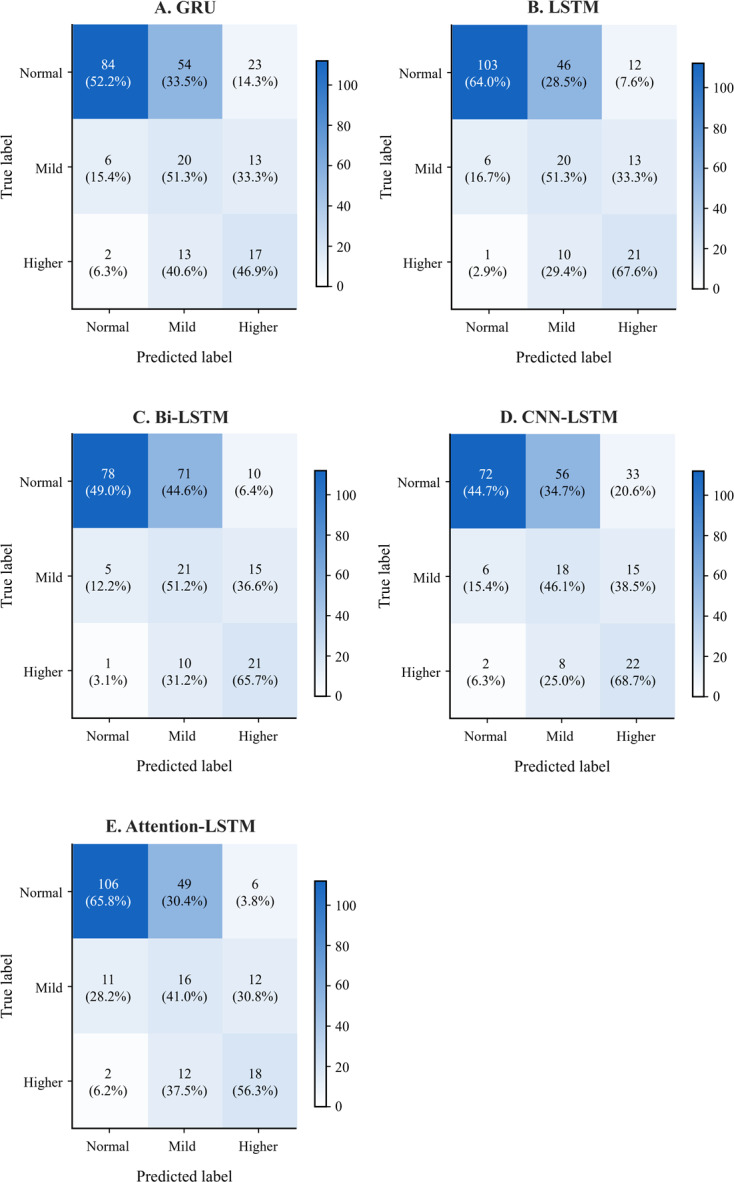
Confusion matrices for depressive symptom classification on the test set. Models evaluated include (A) gated recurrent unit (GRU), (B) long short-term memory (LSTM), (C) bidirectional long short-term memory (Bi-LSTM), (D) convolutional neural network–long short-term memory (CNN-LSTM), and (E) attention-LSTM.

The confusion matrices summarize test-set classification outcomes across 3 depressive symptom groups—normal, mild, and higher—for 5 deep learning models: GRU (Figure 4A), LSTM (Figure 4B), Bi-LSTM (Figure 4C), CNN-LSTM (Figure 4D), and Attention-LSTM (Figure 4E). Recall for the mild symptom group was comparatively limited across all models, with recall ranging from 41% to 51.3%, reflecting frequent misclassification into adjacent categories. For the higher depressive symptom group, CNN-LSTM achieved the highest recall at 68.7% (22/32), followed by Bi-LSTM at 65.7% (21/32) (Figures 4C and 4D). Across all models, misclassification was most prevalent in the mild depressive symptom group, while higher depressive symptoms were least likely to be misclassified as normal across all models.

**Figure 5.**
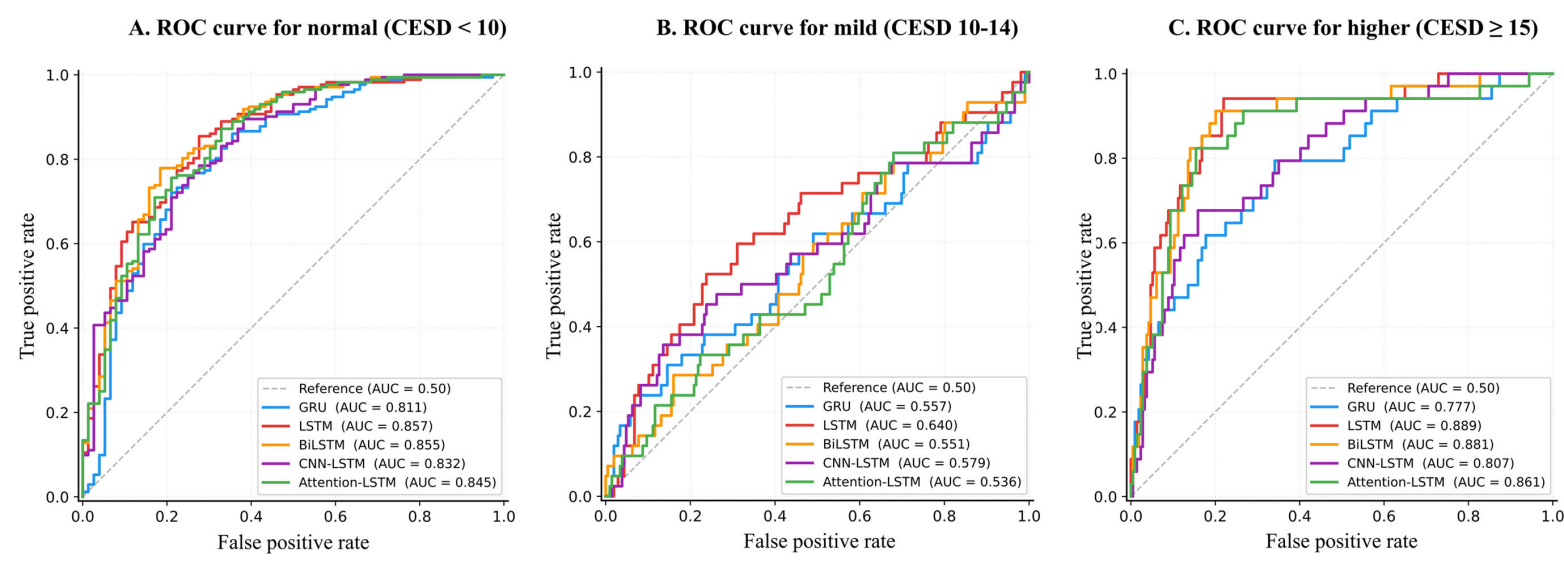
Receiver operating characteristic (ROC) curves for multiclass classification of depressive symptoms across 5 artificial intelligence models (gated recurrent unit [GRU], long short-term memory [LSTM], bidirectional long short-term memory [Bi-LSTM], convolutional neural network–long short-term memory [CNN-LSTM], and attention-LSTM) using a one-versus-rest framework. ROC curves are presented for each depressive symptom group: (A) normal (Center for Epidemiologic Studies Depression scale [CESD] <10), (B) mild (CESD 10-14), and (C) higher (CESD ≥15). Each curve represents the trade-off between the true positive rate (sensitivity) and false positive rate (1 − specificity) for a given model, with the dashed diagonal line indicating reference-level discrimination (area under the curve [AUC]=0.50).

We further evaluated model discrimination using receiver operating characteristic curves across 5 deep learning models (Figure 5). For the normal group, all models achieved strong discrimination, with AUC values ranging from 0.811 to 0.857 (Figure 5A). In contrast, for the mild depressive symptom group (Figure 5B), discriminative performance was substantially lower across all models, with AUC values ranging from 0.536 to 0.640, with LSTM achieving the highest AUC of 0.640, reflecting the inherent challenge of classifying mild depressive symptoms. For the higher depressive symptom group (Figure 5C), models showed improved performance with AUC values ranging from 0.777 to 0.889, with LSTM achieving the highest AUC of 0.889, followed by Bi-LSTM at 0.881 and attention-LSTM at 0.861.

**Figure 6.**
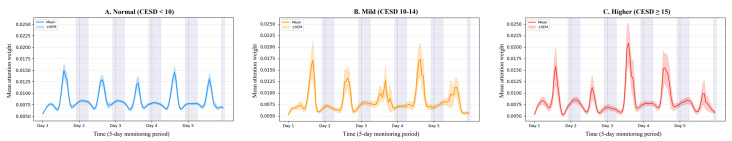
Mean attention weights of the attention-LSTM model across a 5-day monitoring period by depressive symptom group. Mean attention weights are plotted over the 5-day monitoring period for each depressive symptom group. (A) Normal (Center for Epidemiologic Studies Depression scale [CESD] <10), (B) mild (CESD 10-14), and (C) higher (CESD ≥15). Solid lines represent the group-level mean attention weight, and shaded regions indicate ±1 SE of the mean (SEM). Navy-shaded vertical bands denote approximate nighttime periods. The attention-LSTM model consistently assigned higher weights to daytime periods across all groups, reflecting the importance of active-hour behavioral signals in depressive symptom classification.

The attention-LSTM model consistently assigned higher attention weights to daytime periods across all depressive symptom groups throughout the 5-day monitoring period. The normal group demonstrated stable (Figure 6A), rhythmic daily peaks, whereas the mild group showed more variable patterns with pronounced spikes at days 1, 3, and 4 (Figure 6B). The higher group exhibited the largest attention weight peaks, particularly at days 3 and 4, with wider SE of the mean (SEM) bands indicating greater interindividual variability (Figure 6C). Heatmap visualization further confirmed the concentration of attention weights between 12 hours and 16 hours (Figure S9 in Multimedia Appendix 1). To further quantify these patterns, peak attention timepoints were identified for each group (Table S6 and Figure S10 in Multimedia Appendix 1). The normal group peaked at day 1, 14 hours (mean 0.0150, SD 0.0197), the mild group at day 4, 15 hours (mean 0.0175, SD 0.0230), and the higher group showed concentrated peaks on day 3 between 13 hours and 15 hours (mean 0.0181, SD 0.0210), with all 3 peaks occurring within the same day, indicating sustained afternoon behavioral disruption as a key discriminative feature for severe depressive symptoms.

## Discussion

### Principal Findings

This study systematically compared 5 deep learning architectures using multichannel wrist-worn actigraphy to screen for CESD-10–defined depressive symptoms in a propensity score–matched HCHS/SOL cohort. Across both symptom-burden comparisons, discrimination was consistently higher for the higher depressive symptom group than for the mild group. In the mild depressive symptom comparison, LSTM achieved the strongest overall discrimination, with a test-set AUROC of 0.640. In the higher depressive symptom comparison, LSTM also showed the best performance, achieving a test-set AUROC of 0.889, followed closely by Bi-LSTM with an AUROC of 0.881 (Table 5). The confusion matrix findings further indicated fewer missed cases in the higher symptom burden comparison, whereas false-positive classifications remained substantial in both settings (Figure 4). Collectively, these findings suggest that actigraphy-based sequence models may better distinguish higher symptom burden from typical behavioral patterns than mild symptoms. This difference may reflect the more heterogeneous and subtle behavioral signatures associated with mild depressive burden.

From a clinical and public health perspective, these findings support actigraphy-based deep learning as a scalable, passive approach for depressive symptom screening rather than a diagnostic test. In settings where depressive symptoms are underrecognized during routine care, model outputs could complement self-report screening by providing objective, longitudinal behavioral information that may prompt follow-up assessment, particularly among individuals with higher symptom burden.

### Clinical Utility and Implementation Considerations

Classification threshold selection is a key consideration for real-world deployment. In this study, threshold calibration using the Nelder-Mead method on the validation set yielded model-specific optimal thresholds that maximized macro-*F*_1_ performance. Lower thresholds may be preferred in population-level screening to prioritize sensitivity and reduce missed cases, whereas higher thresholds may be more appropriate in resource-limited settings to improve specificity and reduce unnecessary follow-up. As illustrated by the receiver operating characteristic curves (Figure 5), operating points can be adjusted to reflect these trade-offs; however, the optimal threshold should ultimately be determined empirically through prospective validation in the target population.

The confusion matrices (Figure 4) further illuminate the practical implications of these classification outcomes. For the mild depressive symptom group, the attention-LSTM produced 61 false positives and 23 false negatives (Figure 4E), reflecting a greater burden of overidentification than missed cases. For the higher depressive symptom group, despite CNN-LSTM achieving the highest recall, the Bi-LSTM yielded 11 false negatives with a false-positive rate of 12.5% (n=25), suggesting a more favorable trade-off between sensitivity and specificity (Figure 4C). These findings highlight a key characteristic of screening-oriented models: while sensitivity can be improved, false-positive rates may remain substantial, especially for the mild depressive symptom group.

In real-world clinical settings, false-positive predictions may increase patient burden, clinician workload, and health care costs. To address this, we propose a stepped implementation pathway in which a positive actigraphy-based screen is followed by a low-burden secondary assessment, such as a validated Patient Health Questionnaire-9 (PHQ-9) [42], rather than immediate referral for specialist evaluation. Repeating actigraphy-based screening over time may further help confirm persistent behavioral patterns before clinical referral. Individuals who screen positive across both stages could then be prioritized for formal clinical assessment by a mental health professional. The proposed model is not intended to replace clinical diagnosis but to function as a complementary screening signal within existing care pathways, enabling earlier identification in settings where depressive symptoms remain underrecognized during routine care.

### Comparison With Prior Work

Prior studies using smartphones and consumer wearables have reported predictive value for depressive outcomes; however, as discussed in the Introduction section, these modalities differ fundamentally from research-grade actigraphy in sensor standardization, algorithmic transparency, and signal continuity, limiting direct comparisons. Among smartphone-based approaches, Saeb et al [13] demonstrated that GPS-derived features, including location variance and circadian movement, were significantly correlated with PHQ-9 depressive symptom severity (|r|≥ 0.43). Ikäheimonen et al [14] showed that smartphone behavioral features, including screen-off events, communication patterns, and location data, classified depressive states with an accuracy of 82% (95% CI 80%‐84%) in a clinically diagnosed sample. Consumer wearable studies have leveraged heart rate variability and circadian rhythm features for depressive symptom screening; for instance, Rykov et al [11] used Fitbit-derived features and reported an accuracy of 0.80, though performance varied across subgroups, reflecting the influence of population characteristics and feature selection on model generalizability.

Beyond modality, this study also differs from prior actigraphy work in its modeling approach. Incorporating multi-wavelength light exposure alongside activity and wake status offers a richer behavioral representation than studies relying on activity alone. Performance differences across model architectures were not uniform, suggesting that model selection should align with the intended screening target and the acceptable trade-off between missed cases and false alarms.

Table 6 summarizes representative studies that differ in outcome definitions, populations, and input features. Notably, all prior studies employed binary classification frameworks, whereas the present study simultaneously classified three depressive symptom groups, which inherently increases task complexity and limits direct performance comparison. Nonetheless, these studies provide useful context, as the present results extend prior evidence that AI models can support passive screening of depressive symptoms from wearable data. Specifically, Vahedifard et al [23] presented engineered actigraphy-based activity features using XGBoost for 4-class classification of inpatient adolescents with bipolar disorder. Price et al [24] applied a Gramian angular field CNN approach and highlighted the informativeness of nighttime motor patterns, although sensitivity was comparatively lower, potentially reflecting differences in feature scope and labeling strategy.

**Table 6.** Performance comparison of actigraphy-based models for 10-item Center for Epidemiologic Studies Depression scale defined depressive symptom screening.

Research work	Count, n^a^	Models	Class	Accuracy	Sensitivity	Specificity	AUC^b^
Jacobsen et al 2020 [43]	178	CNN^c^	Binary	0.73	0.65	0.78	0.83
Rykov et al 2021 [11]	267	XGBoost^d^	Binary	0.80	0.82	0.78	0.75
Kim et al 2021 [44]	47	LR^e^	Binary	0.91	0.88	0.94	0.96
Ghate et al 2023 [45]	59	LOOCV^f^	Binary	0.79	0.59	0.86	—^u^
Chen et al 2023 [46]	80	RF^h^ and KNN^i^	Binary	0.66	0.67	0.68	0.68
Lee et al 2024 [26]	352	ResNet	Binary	0.88	0.83	0.91	0.94
Price et al 2024 [27]	1232	Transf^s^	Binary	0.83	0.80	0.86	0.89
Price et al 2024 [24]	8378	CNN-GAF^t^	Binary	—	0.45	0.82	0.68
Our study, 2026	1160	LSTM^r^	3-class^m^	0.75	0.60	0.83	0.80

a n is the number of participants included in the analysis.

b AUC: area under the curve.

c CNN: convolutional neural network.

d XGBoost: Extreme Gradient Boosting.

e LR: linear regression.

f LOOCV: leave-one-out cross-validation.

g Not available.

h RF: random forest.

i KNN: k-nearest neighbors.

j Transf: transfer learning.

k CNN-GAF: convolutional neural network–Gramian angular field.

l LSTM: long short-term memory.

m Values represent macro-averaged one-versus-rest performance metrics across three classes.

Prior work has evaluated deep learning approaches for depression screening using either actigraphy as a stand-alone modality or in combination with complementary modalities (Table 6). Lee et al [26] implemented a mixed-input framework integrating actigraphy-derived step counts with sleep stage information and survey measures, reporting an AUC of 0.94. Price et al [27] presented transformer-based architectures to model long-range temporal dependencies in actigraphy sequences, yielding an AUC of 0.89. Jacobsen et al [43] used CNN-based models to distinguish individuals with and without depression, reporting an accuracy of 0.73.

Additional studies have incorporated transfer learning and real-world wearable platforms. Ghate et al [45] proposed a real-time screening pipeline using transfer learning on Fitbit data, achieving 79% accuracy with leave-one-out cross-validation in a small sample. Rykov et al [11] evaluated an XGBoost (Extreme Gradient Boosting)–based machine learning model using wearable-derived digital biomarkers, such as circadian rhythm regularity and nighttime heart rate variation, for binary classification of depressive symptom status in a contrasted subsample, achieving an AUC of 0.75.

### Strengths and Limitations

Unlike studies that rely primarily on engineered features or small-sample transfer-learning settings, we trained 5 deep learning models directly on raw multichannel actigraphy signals in a large, ethnically diverse Hispanic/Latino community cohort (n=1160), a population that remains underrepresented in actigraphy-based mental health research. All models used relatively lightweight architectures ranging from 86,817 to 518,065 parameters, supporting computational feasibility for real-world deployment. We further characterized symptom burden across three depressive symptom groups and reported complementary discrimination and error-profile metrics to quantify trade-offs between missed cases and false alarms. The use of 6 synchronized actigraphy channels, including activity counts, wake status, and multiwavelength light exposure, provided a richer behavioral representation than studies relying on single-channel inputs.

Several limitations should be considered. First, depressive symptom status was defined using the CESD-10, a self-reported screening tool rather than a structured clinical diagnosis; therefore, our models estimate symptom burden, not clinician-diagnosed major depressive disorder. Second, actigraphy nonwear and missing segments may introduce residual measurement noise despite quality-control procedures. Third, external validation in independent cohorts is needed to establish transportability across diverse populations. Fourth, attention weight patterns in the attention-LSTM reflect selective temporal weighting rather than formal clinical explanations; individual-level attribution methods, such as Shapley Additive Explanations–based approaches, represent an important direction for future work.

### Future Research

Future studies should validate these findings using clinically adjudicated outcomes across diverse subgroups and comorbid conditions. Longitudinal monitoring designs should be explored to assess whether actigraphy-derived behavioral signatures can track symptom trajectories and detect early relapses or recovery patterns. Additionally, the generalizability of models trained on research-grade actigraphy to consumer wearables and smartphone-based platforms warrants investigation. Establishing interpretable and externally validated models will be essential for translating actigraphy-based screening into real-world mental health workflows.

### Conclusions

In a matched Hispanic/Latino community-based cohort, deep learning models trained on multichannel wrist-worn actigraphy demonstrated stronger discrimination for higher depressive symptoms than for mild depressive symptoms, with LSTM achieving the strongest overall performance and a macro-averaged AUROC of 0.80. These findings suggest that actigraphy-derived data can support AI-based passive screening of depressive symptoms; however, false-positive rates remain notable, and external validation and improved interpretability are needed before clinical deployment. An actigraphy-based AI model may serve as a scalable, passive, and noninvasive complementary signal to aid early screening alongside traditional depressive symptom assessments.

## Supplementary material

10.2196/91479 Multimedia Appendix 1 Detailed model descriptions and architectures (gated recurrent unit, long short-term memory, bidirectional long short-term memory, convolutional neural network–long short-term memory, and attention-long short-term memory), layer-wise specifications, multiclass performance metrics, and attention-based long short-term memory weight analyses including heatmaps and peak timepoint distributions.

## Abbreviations

AI: artificial intelligence
AUC: area under the curve
AUROC: area under the receiver operating characteristic curve
Bi-LSTM: bidirectional long short-term memory
CESD-10: 10-item Center for Epidemiologic Studies Depression scale
CNN: convolutional neural network
CNN-LSTM: convolutional neural network–long short-term memory
GRU: gated recurrent unit
HCHS/SOL: Hispanic Community Health Study/Study of Latinos
HS/GED: High school / General Educational Development
L5: average activity within the least active 5-hour period of the day
LSTM: long short-term memory
M10: average activity within the most active 10-hour period of the day
NSRR: National Sleep Research Resource
PHQ-9: Patient Health Questionnaire-9
XGBoost: Extreme Gradient Boosting
