# Supplementary material for: Passive Screening for Depressive Symptoms Using Daily Wrist Actigraphy and Deep Learning: Model Development and Validation Study
Source: JMIR Mhealth Uhealth. 2026 Jul 15;14:e91479. doi: 10.2196/91479 (PMC13372263; doi:10.2196/91479)
Supplement: Multimedia Appendix 1 [file mhealth-v14-e91479-s001.docx]

**Figure S1.** Study participant flow chart.

####

**Table S1.** Multi-class AI model performance across training, validation, and test sets for depressive symptom screening.

| **Models** | **Data set** | **Normal (CESD < 10)** | | | **Mild (CESD 10-14)** | | | **Higher (CESD ≥ 15)** | | |
| --- | --- | --- | --- | --- | --- | --- | --- | --- | --- | --- |
|  |  | **Acc** | **F1** | **AUC** | **Acc** | **F1** | **AUC** | **Acc** | **F1** | **AUC** |
| **Single AI models** | | | | | | | | | | |
| **GRU** | Train | 0.759 | 0.668 | 0.857 | 0.767 | 0.642 | 0.848 | 0.843 | 0.629 | 0.916 |
|  | Valid | 0.625 | 0.672 | 0.744 | 0.665 | 0.425 | 0.523 | 0.798 | 0.537 | 0.762 |
|  | Test | 0.634 | 0.668 | 0.811 | 0.639 | 0.418 | 0.557 | 0.786 | 0.476 | 0.777 |
|  |  |  |  |  |  |  |  |  |  |  |
| **LSTM** | Train | 0.721 | 0.655 | 0.811 | 0.637 | 0.442 | 0.694 | 0.791 | 0.632 | 0.829 |
|  | Valid | 0.673 | 0.719 | 0.782 | 0.645 | 0.463 | 0.573 | 0.859 | 0.557 | 0.810 |
|  | Test | 0.726 | 0.767 | 0.857 | 0.682 | 0.437 | 0.640 | 0.851 | 0.554 | 0.889 |
|  | | | | | | | | | | |
| **Bi-LSTM** | Train | 0.710 | 0.565 | 0.818 | 0.599 | 0.475 | 0.694 | 0.796 | 0.641 | 0.845 |
|  | Valid | 0.573 | 0.586 | 0.782 | 0.524 | 0.401 | 0.573 | 0.863 | 0.564 | 0.822 |
|  | Test | 0.625 | 0.644 | 0.855 | 0.576 | 0.414 | 0.551 | 0.855 | 0.571 | 0.881 |
| **Hybrid AI models** | | | | | | | | | | |
| **CNN LSTM** | Train | 0.707 | 0.564 | 0.812 | 0.720 | 0.532 | 0.766 | 0.769 | 0.684 | 0.893 |
|  | Valid | 0.561 | 0.582 | 0.766 | 0.661 | 0.473 | 0.508 | 0.730 | 0.446 | 0.817 |
|  | Test | 0.597 | 0.615 | 0.832 | 0.629 | 0.402 | 0.579 | 0.758 | 0.434 | 0.807 |
|  |  |  |  |  |  |  |  |  |  |  |
| **Attention LSTM** | Train | 0.729 | 0.668 | 0.805 | 0.632 | 0.436 | 0.664 | 0.796 | 0.615 | 0.841 |
|  | Valid | 0.682 | 0.738 | 0.771 | 0.669 | 0.459 | 0.621 | 0.891 | 0.597 | 0.826 |
|  | Test | 0.706 | 0.756 | 0.845 | 0.645 | 0.418 | 0.536 | 0.859 | 0.533 | 0.861 |

*Note****:*** Accuracy, F1 score, and AUC were computed using a one-versus-rest framework, in which each depressive symptom group was treated as the positive class against the remaining two groups combined.

#### Gated-Recurrent Memory

The gated recurrent unit (GRU) is a streamlined recurrent neural network (RNN) model designed to model temporal dependencies in sequential data while mitigating vanishing gradient effects. GRUs are effective for time-series such as wrist actigraphy, where behavioral patterns evolve. In this study, GRU layers were stacked and combined with batch normalization to improve temporal feature learning (**Table 1**). Unlike long short-term memory (LSTM) networks, GRUs do not use a separate memory cell. Instead, they regulate information flow using an update gate $z_{t}$and a reset gate $r_{t}$(**Figure 1A**):

z_t_ = σ (W^xz^x_t_ + W^hz^h_t−1_ + b_z_)

r_t_ = σ (W^xr^x_t_ + W^hr^h_t−1_ + b_r_)

The hidden state of the candidate $\hat{h}_{t}$is computed as:

$\hat{h}_{t}$ = tanh (Wx_t_ + Wh_t−1_ × r_t_)

The final hidden state $h_{t}$is then obtained by interpolating between the previous hidden state and the candidate state:

h_t_ = (1 − z_t_ ) × h_t-1_ + z_t_ × $\hat{h}_{t}$

**Figure S2.** Schematic diagram of the GRU model architecture. Multichannel actigraphy signals are normalized using batch normalization prior to input. The model comprises two stacked GRU layers, with batch normalization and dropout applied between layers. The final sequence representation is flattened and passed through a fully connected SoftMax output layer to produce a three-class probability distribution across normal, mild, and higher depressive symptom groups.

#### Long short-term memory

Long short-term memory (LSTM) networks are recurrent neural networks that capture long-range temporal dependencies using an explicit memory cell, which helps mitigate vanishing-gradient issues. This is beneficial for actigraphy sequences where patterns may reflect both short-term fluctuations and longer-term circadian trends (Table 1). An LSTM unit updates its cell state $c_{t}$and hidden state $h_{t}$through input, forget, and output gates (**Figure 1B**):

i_t_ (input gate) = σ (W^i^x_t_ + U^i^h_t−1_ + b_i_)

f_t_ (forger gate)= σ (W^f^x_t_ + U^f^h_t−1_ + b_f_)

o_t_ (output gate)= σ (W^o^x_t_ + U^o^h_t−1_ + b_o_)

g_t_ (candidate cell state)= tanh (W^g^x_t_ + U^g^h_t−1_ + b_c_)

c_t_ (cell state update)= f_t_ × c_t−1_ + i_t_ × g_t_

h_t_ (hidden state output)= o_t_ × tanh (c_t_)

Here, $x_{t}$denotes the input at time step $t$, $h_{t}$the hidden state, and $\sigma(\cdot)$the sigmoid activation.

**Figure S3.** Schematic diagram of the LSTM model architecture. Multichannel actigraphy signals are normalized using batch normalization prior to input. The model comprises two stacked LSTM layers, with batch normalization and dropout applied between layers. The final sequence representation is flattened and passed through a fully connected SoftMax output layer to produce a three-class probability distribution.

**Figure S4.** Architectural overview of gated recurrent unit (GRU) and long short-term memory (LSTM) networks. **A.** The GRU uses reset and update gates**. B.** The LSTM employs forget, input, and output gates, and a memory cell state.

**Table S2.** Layer-wise architecture specifications of the GRU and LSTM models.

| **Layers** | **GRU** | | | **LSTM** | | |
| --- | --- | --- | --- | --- | --- | --- |
|  | Memory cells | Activation | Parameter count | Memory cells | Activation | Parameter count |
| batchnorm_0 | — | — | 64 | — | — | 64 |
| rnn_1 | 60 | tanh/SoftMax | 20,160 | 60 | tanh/SoftMax | 29,760 |
| batchnorm_1 | — | — | 120 | — | — | 120 |
| rnn_2 | 80 | tanh/SoftMax | 31,680 | 80 | tanh/SoftMax | 45,120 |
| batchnorm_2 | — | — | 160 | — | — | 160 |
| rnn_3 | 100 | tanh/SoftMax | 50,400 | 100 | tanh/SoftMax | 72,400 |
| batchnorm_3 | — | — | 200 | — | — | 200 |
| rnn_4 | 120 | tanh/SoftMax | 67,680 | 120 | tanh/SoftMax | 110,880 |
| batchnorm_4 | — | — | 240 | — | — | 240 |
| dense_1 | 1 | tanh/SoftMax | 121 | 1 | tanh/SoftMax | 121 |
| **Total** |  |  | 170,345 |  |  | 258,585 |

*Note****:*** GRU and LSTM models used identical layer structures. Batch normalization was applied after each recurrent layer, and a SoftMax activation was used in the output layer. Parameter counts reflect model complexity.

#### Bidirectional LSTM

Bidirectional LSTM (BiLSTM) processes the input sequence in both forward and backward directions and concatenates the resulting hidden states, enabling the model to leverage contextual information from both past and future time steps (**Figure S5**):

$\vec{h_{t}}$ = LSTM_forward_ (x_t_, $\vec{h_{t-1}}$)

$h_{t}$ = LSTM_backward_ (x_t_, $h_{t+1})$

h_t_ = [ $\vec{h_{t}}$ ; $h_{t}$ ]

This bidirectional representation can improve actigraphy-based classification by capturing richer temporal dependencies associated with depressive symptom patterns (**Figure S6**).

**Figure S5.** Architectural diagram of bidirectional long short-term memory (BiLSTM). The input sequence is processed in parallel by forward and backward LSTM layers, and hidden representations from both directions are concatenated for classification.

**Figure S6.** Schematic diagram of the Bi-LSTM model architecture. Multichannel actigraphy signals are normalized using batch normalization prior to input. The bidirectional LSTM processes each sequence in both forward and backward directions, with batch normalization and dropout applied thereafter. The concatenated forward and backward representations are flattened and passed through a fully connected SoftMax output layer.

**Table S3.** Layer-wise architecture specifications of the Bi–LSTM model

| **Layers** | **Memory cells** | **Activation** | **Output Shape** | **Parameters** |
| --- | --- | --- | --- | --- |
| batchnorm_0 | — | — | 60 × 6 | 64 |
| bi_lstm_1 | 60 | tanh/ SoftMax | 60 × 120 | 59,520 |
| batchnorm_1 | — | — | 60 × 240 | 240 |
| bi_lstm_2 | 80 | tanh/ SoftMax | 60 × 240 | 90,240 |
| batchnorm_2 | — | — | 60 × 320 | 320 |
| bi_lstm_3 | 100 | tanh/ SoftMax | 60 × 320 | 144,800 |
| batchnorm_3 | — | — | 60 × 400 | 400 |
| bi_lstm_4 | 120 | tanh/ SoftMax | 400 | 221,760 |
| batchnorm_4 | — | — | 480 | 480 |
| dense_1 | 1 | SoftMax | 1 | 241 |
| Total |  | — |  | 518,065 |

*Note*: The Bi-LSTM processes sequences in both forward and backward directions, resulting in doubled feature representations. Batch normalization was applied after each bidirectional layer, and SoftMax activation was used in the output layer. Parameter counts reflect model complexity.

#### 1D CNN–LSTM model

The hybrid 1D CNN–LSTM architecture combines convolutional feature extraction with recurrent temporal modeling. The 1D CNN layers learn local patterns from actigraphy sequences, including short-term activity fluctuations, using convolution and pooling operations, producing compact feature representation. These features are then passed to an LSTM layer to capture longer-range temporal dependencies across the sequence. This design enables the model to jointly learn discriminative local features and temporal dynamics for binary classification (**Figure S7**).

**Figure S7.** Schematic diagram of the CNN–LSTM model architecture. Multichannel actigraphy signals are normalized using batch normalization prior to input. Three 1D convolutional blocks with increasing filter sizes extract local temporal features, followed by batch normalization and dropout. The resulting feature maps are passed to an LSTM layer to model longer-range temporal dependencies, and predictions are generated through a fully connected SoftMax output layer.

**Table S4.** Layer-wise architecture specifications of the 1D CNN–LSTM model.

| **Layers** | **Filter Size / Operation** | **Activation** | **Output Shape** | **Parameters** |
| --- | --- | --- | --- | --- |
| batchnorm_1 | — | — | 60 × 32 | 64 |
| conv1 | 32 × 11 | ReLU | 60 × 32 | 2,144 |
| maxpool_1 | 2 × 1 | — | 30 × 32 | — |
| conv2 | 64 × 5 | ReLU | 30 × 64 | 10,304 |
| batchnorm_2 | — | — | 30 × 64 | 128 |
| maxpool_2 | 2 × 1 | — | 15 × 64 | — |
| conv3 | 128 × 3 | ReLU | 15 × 128 | 24,704 |
| maxpool_3 | 2 × 1 | — | 7 × 128 | — |
| lstm | 64 (hidden units) | SoftMax | 64 | 49,408 |
| dropout | 0.5 | — | 64 | — |
| dense_2 | 64 → 1 | SoftMax | 1 | 65 |
| Total | 224 filters |  |  | 86,817 |

Note: ReLU activation was applied to convolutional layers. SoftMax activation was used in the LSTM and output layer. “64→1” indicates the mapping from the LSTM output to a single-node output.

#### Attention–LSTM model

**Figure S8.** Schematic diagram of Attention-LSTM model architecture. Multichannel actigraphy signals are normalized using batch normalization prior to input. An LSTM layer models temporal dependencies in the sequence, followed by a single-head attention module that emphasizes the most informative time steps. The attended representations are passed through dropout and a fully connected SoftMax output layer to produce a three-class probability distribution.

**Table S5.** Layer-wise architecture of the Attention-LSTM model.

| **Layers** | **Units / Operation** | **Activation** | **Output Shape** | **Parameters** |
| --- | --- | --- | --- | --- |
| batchnorm_1 | — | — | 60 × 32 | 64 |
| conv1 | 32 × 11 | ReLU | 60 × 32 | 2,144 |
| maxpool_1 | 2 × 1 | — | 30 × 32 | — |
| conv2 | 64 × 5 | ReLU | 30 × 64 | 10,304 |
| batchnorm_2 | — | — | 30 × 64 | 128 |
| maxpool_2 | 2 × 1 | — | 15 × 64 | — |
| conv3 | 128 × 3 | ReLU | 15 × 128 | 24,704 |
| maxpool_3 | 2 × 1 | — | 7 × 128 | — |
| attention | single head (128d) | — | 7 × 128 | 49,280 |
| lstm | 64 (hidden units) | SoftMax | 64 | 49,408 |
| dropout | 0.5 | — | 64 | — |
| dense_2 | 64 → 1 | SoftMax | 1 | 65 |
| Total | 224 filters |  |  | 136,097 |

Note***:*** ReLU activation was applied to convolutional layers. The attention module uses scaled dot-product attention over temporal features. SoftMax activation was used in the LSTM and output layer. “64→1” denotes the mapping from the LSTM output to a single-node output.

**Figure S9.** Heatmap of mean Attention weights by hour of day and monitoring day across depressive symptom groups. Mean attention weights derived from the Attention-LSTM model are displayed as a function of hour of day (x-axis, 00h–23h) and monitoring day (y-axis, Day 1–Day 5) for each depressive symptom group: **(A)** Normal (CESD < 10; n = 580), **(B)** Mild (CESD 10–14; n = 318), and **(C)** Higher (CESD ≥ 15; n = 262). Color intensity represents the magnitude of mean attention weight, with darker shades indicating timepoints to which the model assigned greater importance during classification. White stars (*) denote the peak attention timepoint within each group. The Normal group exhibited peak attention on Day 1, 14:00h, reflecting early afternoon activity patterns in the first monitoring day. The Mild group showed a delayed peak at Day 4, 15:00h, suggesting the model prioritized mid-to-late monitoring period behavioral signals. The Higher group demonstrated the highest overall attention weights with a peak at Day 3, 14:00h and broader temporal distribution across multiple days, indicating more diffuse and sustained behavioral patterns associated with greater depressive symptom severity. Across all groups, attention weights were consistently elevated during the afternoon window (12:00h–16:00h), suggesting that post-noon behavioral signals carry the greatest discriminative value for depressive symptom classification.

**Table S6.** Top 3 Peak Attention Timepoints per Depressive Symptom Group (Attention-LSTM, 5-Day Monitoring)

| **Group** | **Rank** | **Day** | **Hour** | **Mean Attention Weight** |
| --- | --- | --- | --- | --- |
| Normal (CESD < 10) | 1 | 1 | 14:00 | 0.01498 |
|  | 2 | 4 | 15:00 | 0.01448 |
|  | 3 | 1 | 15:00 | 0.01372 |
| Mild (CESD 10–14) | 1 | 4 | 15:00 | 0.01747 |
|  | 2 | 1 | 16:00 | 0.01719 |
|  | 3 | 4 | 14:00 | 0.01679 |
| Higher (CESD ≥ 15) | 1 | 3 | 14:00 | 0.02096 |
|  | 2 | 3 | 13:00 | 0.01981 |
|  | 3 | 3 | 15:00 | 0.01795 |

*Note****:*** Peak attention timepoints were identified from the Attention-LSTM model's attention weight distributions averaged across participants within each group.

**Figure S10.** Comparison of mean attention weights across depressive symptom groups over the 5-day monitoring period. Mean attention weights derived from the Attention-LSTM model are plotted as a function of time for three depressive symptom groups: Normal (blue), Mild depressive symptoms (orange), and Higher depressive symptoms (red). Dashed vertical lines indicate day boundaries across the 5-day monitoring period. The Higher depressive symptoms group demonstrated the largest attention weight peaks, particularly at Days 1, 3, and 4, with a pronounced spike at Day 3 reaching approximately 0.021. The Mild depressive symptoms group exhibited elevated peaks at Days 1, 2, and 4, while the Normal group showed moderate, rhythmic peaks throughout the monitoring period. All three groups demonstrated consistent daytime activity patterns with attention weights converging to baseline levels during low-activity periods. This overlay plot complements Figure 6 by enabling direct visual comparison of temporal attention dynamics across depressive symptom severity groups.

### Abbreviations

GRU: gated recurrent unit
LSTM: long short-term memory
BiLSTM: bidirectional long short-term memory
RNN: recurrent neural network
